# Cost Effectiveness of the 8-Strain Probiotic in Primary and Secondary Prophylaxis of Pouchitis

**DOI:** 10.1016/j.gastha.2025.100776

**Published:** 2025-08-28

**Authors:** Gaurav Syal, Siddharth Singh, Edward L. Barnes

**Affiliations:** 1 Vatche and Tamar Manoukian Division of Digestive Diseases, University of California Los Angeles, Los Angeles, California; 2 Division of Gastroenterology and Hepatology, University of California San Diego, San Diego, California; 3 Division of Gastroenterology and Hepatology, University of North Carolina, Chapel Hill, North Carolina

**Keywords:** Cost-Effectiveness, Pouchitis, Prevention, Probiotic

## Abstract

**Background and Aims:**

The 8-strain probiotic formulation appears to be effective for primary and secondary prevention of pouchitis in patients with ulcerative colitis after ileal pouch anal anastomosis. We aimed to study its cost-effectiveness compared to no prophylaxis in these settings.

**Methods:**

We constructed decision trees with Markov models for primary prevention of pouchitis and secondary prevention of relapsing pouchitis in patients with ulcerative colitis after ileal pouch anal anastomosis. All patients were followed for 2 years. In the primary prophylaxis model, Markov cycle length was 2 weeks and the pouchitis treatment sequence was ciprofloxacin, metronidazole and a combination of ciprofloxacin and tinidazole. In the secondary prophylaxis models, the Markov cycle length was 4 weeks and the pouchitis treatment sequence was ciprofloxacin, metronidazole, ciprofloxacin/tinidazole, vedolizumab and infliximab. Third-party payers’ perspective with a willingness-to-pay threshold of $100,000/quality-adjusted life years (QALYs) was used. Frequent relapsing pouchitis was defined as ≥2 pouchitis episodes/year.

**Results:**

For primary prevention of pouchitis, no prophylaxis was more cost effective compared with the probiotic prophylaxis on base-case analysis (incremental cost effectiveness ratio $236,661/QALY). On base-case analysis for secondary prevention of pouchitis relapse in infrequent pouchitis, no prophylaxis was more cost effective compared to the probiotic prophylaxis (incremental cost effectiveness ratio $153,011/QALY). One-way sensitivity analysis showed that the probiotic prophylaxis would be the dominant strategy in patients with frequent relapsing pouchitis.

**Conclusion:**

Compared to no prophylaxis, the 8-strain probiotic is not cost-effective for primary prevention of pouchitis. It is cost-effective for secondary prophylaxis of frequent pouchitis but not for secondary prophylaxis of infrequent pouchitis.

## Introduction

Pouchitis is the most common inflammatory complication in patients with ulcerative colitis (UC) who undergo restorative proctocolectomy with ileal pouch anal anastomosis (IPAA) with cumulative incidence of around 48% at 2 years and 80% at 30 years.^1^, ^2^, ^3^ Most patients who develop pouchitis experience a single episode that improves with a short course of antibiotics. However, many patients develop recurrent or relapsing pouchitis and 17% progress to a chronic form of pouchitis that can be antibiotic-dependent or antibiotic refractory.^4^

Given its high incidence and significant burden, prevention of pouchitis is an attractive approach.^5^^,^^6^ Since pouchitis may be mediated by gut dysbiosis, several studies have evaluated the effectiveness of probiotics in prevention of pouchitis. A placebo-controlled randomized controlled trial (RCT) showed that daily use of an 8-strain combination of *Lactobacillus paracasei* subsp *paracasei*, *L. plantarum, L. acidophilus, L. delbrueckii* subspecies *bulgaricus, Bifidobacterium longum* subspecies *longum, B. breve, B. longum* subspecies *infantis, and Streptococcus salivarius* subspecies *thermophilus*, is effective in primary prevention of pouchitis.^7^ This probiotic was also found to be effective for secondary prevention of pouchitis (ie, preventing relapse) in patients with recurrent pouchitis in three placebo-controlled RCTs.^8^, ^9^, ^10^ Despite its effectiveness, the cost of daily use of the 8-strain probiotic formulation can be significant and the overall cost-effectiveness of this prophylactic strategy has not been evaluated. This knowledge can help the third-party payers and policy makers evaluate whether allocation of resources towards this strategy can help maximize health outcomes in patients with IPAA within the budgetary constraints.

Hence, we evaluated the cost-effectiveness of prophylaxis with the 8-strain probiotic in primary and secondary prevention of pouchitis in patients with UC who undergo IPAA compared with no prophylaxis.

## Methods

### Model Specifications

We used a decision analysis software (TreeAge Pro Healthcare, version 2020, Williamstown, MA) to evaluate the cost-effectiveness of the 8-strain probiotic for primary and secondary prevention of pouchitis in a hypothetical cohort of individuals who have undergone colectomy with IPAA for UC from a third-party payer perspective. For the base case analyses, we assumed an adult male with a body weight of 70 kg.

#### Primary prophylaxis

**Figure 1:**
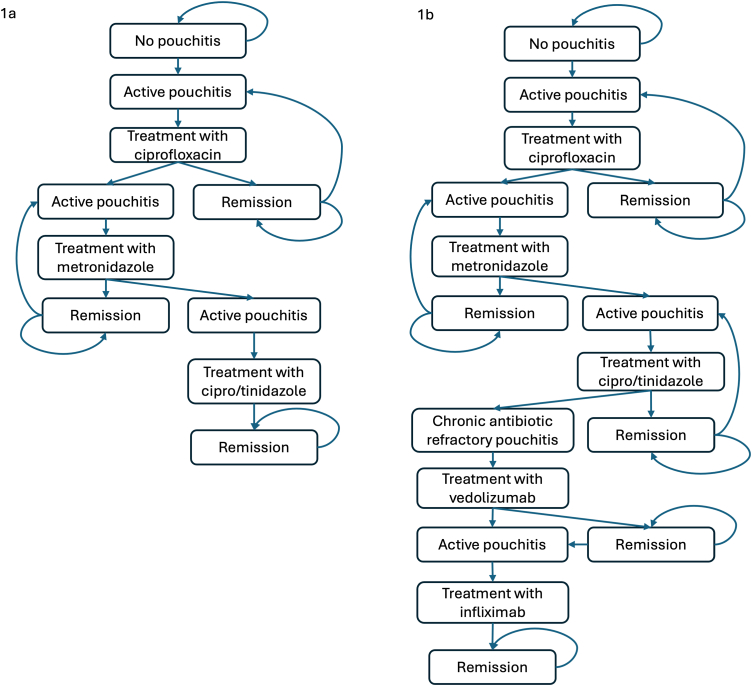
Markov models for primary prevention (A) and secondary prevention (B) of pouchitis.

Individuals with UC who underwent IPAA, and never experienced pouchitis entered the decision tree and were assigned to either prophylaxis with the 8-strain probiotic or no prophylaxis. This cohort was followed over 2 years through a series of Markov cycles representing transitions between different health states (Figure 1A). We selected a Markov cycle length of 2 weeks to match the typical duration of antibiotic therapy for acute pouchitis. The truncated cost-effectiveness model is shown in Figure A1.

At the time of the first pouchitis episode, patients were assumed to undergo evaluation including an outpatient provider visit, a pouchoscopy under moderate sedation and laboratory workup including complete blood count (CBC), comprehensive metabolic panel, C-reactive protein and stool *Clostridioides difficile* (*C. difficile*) testing. After the workup, they were treated with a 2-week course of ciprofloxacin 500 mg twice daily. Those who did not improve with ciprofloxacin were treated with metronidazole 500 mg twice daily for 2 weeks and metronidazole nonresponders were treated with a combination of ciprofloxacin 500 mg twice daily and tinidazole 500 mg twice daily for 4 weeks. To account for variations in clinical practice related to diagnosis and treatment of pouchitis, we also performed an additional base-case analysis assuming that the first pouchitis episode was treated empirically with ciprofloxacin and the workup, including laboratory tests, stool tests and pouchoscopy, was only performed when patients developed recurrent or antibiotic refractory pouchitis. We assumed a 100% response rate with combination antibiotic regimen in the main analysis since we anticipated that a very small percentage of the base case cohort would reach the metronidazole refractory pouchitis state in our model and that extending the model further would not significantly impact the results. However, to confirm our assumption, we also performed extended analysis where nonresponders to combination antibiotics were treated with vedolizumab (VDZ) and those with VDZ nonresponse or loss of response were treated with infliximab (IFX) with a 100% assumed response rate. In patients who experienced pouchitis, subsequent pouchitis episodes were empirically treated with a 2-week course of the antibiotic that was effective for their previous pouchitis episode. We assumed that patients on the 8-strain probiotic prophylaxis who remained pouchitis-free continued it and those who developed pouchitis discontinued it due to lack of efficacy.

#### Secondary prophylaxis

Individuals with UC-IPAA and relapsing pouchitis entered the decision tree in remission and were assigned to either prophylaxis with the 8-strain probiotic or no prophylaxis. This cohort was followed over 2 years through a series of Markov cycles (Figure 1B). We chose a Markov cycle length of 4 weeks to match the 4-week course of antibiotic that is frequently used to treat recurrent pouchitis. The truncated cost-effectiveness model is shown in Figure A2.

Since these patients had previously confirmed pouchitis, pouchitis relapses were treated empirically with antibiotic courses without any workup. The first relapse was treated with a 4-week course of ciprofloxacin 500 mg twice daily. Ciprofloxacin nonresponders were treated with metronidazole 500 mg twice daily for 4 weeks and metronidazole nonresponders were treated with a 4-week course of ciprofloxacin 500 mg and tinidazole 500 mg twice daily. Subsequent pouchitis relapses were treated with an empirical 4-week course of the same antibiotic that was effective in treating the previous episode. Nonresponders to dual antibiotics were assumed to have chronic antibiotic refractory pouchitis (CARP) and undergo evaluation including an outpatient provider visit and workup including CBC, comprehensive metabolic panel, CRP, stool *C. difficile* testing and a pouchoscopy under moderate sedation. Patients with CARP were treated with VDZ as their first advanced immunosuppressive treatment. In cases of nonresponse or loss of response to VDZ, treatment was changed to IFX. We anticipated that a very small proportion of the base case cohort will transition to the VDZ refractory CARP state and extending the model further would not impact the results. Hence, we chose to terminate the model with an assumed 100% response rate to IFX. Patients on the 8-strain probiotic were assumed to continue it regardless of pouchitis relapses because in clinical practice, prophylaxis is often continued despite pouchitis relapses with the goal of reducing the frequency of relapses. However, patients who progressed to develop CARP were assumed to discontinue the probiotic prohylaxis.

### Clinical Probability Estimates

Our base-case model incorporated various probability estimates relevant to pouchitis (Tables 1 and 2). For primary prophylaxis, we used 10% annual probability of developing pouchitis on the 8-strain probiotic prophylaxis and 40% without any prophylaxis derived from an RCT.^7^ For secondary prophylaxis, we used a 14% annual pouchitis relapse rates on the 8-strain probiotic obtained from a meta-analysis of RCTs and a 39% annual pouchitis relapse rate without prophylaxis derived from a large observational study.^1^^,^^11^ Probability estimates for treatment response to antibiotics and advanced therapies were obtained from the American Gastroenterological Association (AGA) clinical guidelines on management of pouchitis^11^ and probability of loss of response to VDZ was derived from the published literature.^12^

**Table 1:** Probability, Cost, and Utility Estimates Used in the Base Case Analysis and Monte Carlo Analyses for Primary Prophylaxis of Pouchitis

Description	Base-case value	Range for Monte Carlo analysis	References
Probability of first pouchitis on the 8-strain probiotic prophylaxis in 1 y	0.1	0.05–0.25	^7^
Probability of first pouchitis on no prophylaxis in 1 y	0.4	0.25–0.50	^7^
Probability of recurrent pouchitis in 1 y after index pouchitis episode	0.39	0.20–0.50	^21^
Probability of response to a 2-wk course of ciprofloxacin	0.77	0.60–0.95	^11^
Probability of response to a 2-wk course of metronidazole	0.70	0.55–0.90	^11^
Cost of the 8-strain probiotic 900 billion strains daily for 2 wk	$87.2	$50–$150	^17^
Cost of ciprofloxacin 500 mg twice daily for 2 wk	$12	$6–$24	^16^
Cost of metronidazole 500 mg twice daily for 2 wk	$22.68	$11–$45	^16^
Cost of ciprofloxacin 500 mg twice daily and tinidazole 500 mg twice daily for 4 wk	$270	$140–500	^16^
Cost of first episode of pouchitis^a^(excluding antibiotic cost)	$612.6	$300–$1000	^14^^,^^15^
Annual utility of no pouchitis	0.91	0.70–0.95	^22^
Annual utility of active pouchitis	0.46	0.30–0.60	^23^

a Includes the cost of a provider visit, laboratory tests, stool *Clostridioides difficile* test and a pouchoscopy under moderate sedation.

**Table 2:** Probability, Cost, and Utility Estimates Used in the Base Case Analysis and Monte Carlo Analyses for Prevention of Pouchitis Relapse

Description	Base-case value	Range for Monte Carlo analysis	References
Probability of pouchitis relapse on the 8-strain probiotic in 1 y	0.14	0.1–0.5	13, 14, 15
Probability of pouchitis relapse in infrequent pouchitis without prophylaxis in 1 y	0.39	0.25–0.55	^1^
Probability of response to 4-wk course of ciprofloxacin	0.77	0.5–0.9	^11^
Probability of response to 4-wk course of metronidazole	0.70	0.5–0.9	^11^
Probability of response to 4-wk course of dual antibiotics	0.69	0.5–0.9	^11^
Probability of response to VDZ	0.52	0.3–0.7	^11^
Probability of response to IFX	1.0	1.0	^11^
Probability of loss of response to VDZ in 1 y	0.40	0.2–0.6	^12^
Cost of the 8-strain probiotic 900 billion daily for 4 wk	174	100–300	^17^
Cost of ciprofloxacin 500 mg twice daily for 4 wk	24	12–48	^16^
Cost of metronidazole 500 mg twice daily for 4 wk	45.4	25–80	^16^
Cost of ciprofloxacin 500 mg twice daily + tinidazole 500 mg twice daily for 4 wk	270	150–500	^16^
Cost of intravenous VDZ 300 mg	6803	3500–10000	^13^
Cost of intravenous IFX 300 mg	987	500–1500	^13^
Cost of evaluation of CARP^a^	128.4	70–200	^14^^,^^15^
Annual utility of no pouchitis	0.91	0.70–0.95	^22^
Annual utility of active pouchitis	0.46	0.30–0.60	^23^

a Includes the cost of a provider visit, laboratory tests, stool *Clostridioides difficile* test and a pouchoscopy under moderate sedation.

Since the pouchitis relapse frequency varies widely among patients with relapsing pouchitis, we evaluated the cost-effectivess of the 8-strain probiotic separately in infrequent and frequent relapsing pouchitis. The frequency of pouchitis relapse in infrequent relapsing pouchitis was derived from a large administrative claims database study that evaluated the probability of pouchitis relapse in average risk patients.^1^ The cost effectiveness of the probiotic prophylaxis in frequent relapsing pouchitis was evaluated using 1-way sensitivity analysis. We used a threshold of ≥2 pouchitis episode per year to define frequent relapsing pouchitis.

### Cost Estimates

The model accounted for the health-care costs in United States dollars from a third-party payers’ perspective. Base-case cost estimates used are shown in Tables 1 and 2. All costs including the cost of drugs, outpatient provider visits and patient evaluation were obtained from the Medicare program.^13^, ^14^, ^15^, ^16^ The only exception was the cost of the 8-strain probiotic that was obtained from the US website of this proprietary product.^17^ Though probiotics are generally not covered by health insurance in the US, we assumed that cost of the 8-strain probiotic was covered by the third-party payer for the purpose of this analysis. Patients were assumed to receive the Food and Drug Administration–approved doses of VDZ and IFX.

### Utility Estimates

Utilities for pouchitis and no pouchitis were expressed as quality-adjusted life years (QALYs) and were obtained from the published literature (Tables 1 and 2). Discounting was performed at a 3% annual rate.

### Outcomes

We used incremental cost effectiveness ratio (ICER) as the main outcome measure for calculating incremental cost per QALY gained among the 2 competing strategies. We used $100,000 per QALY as the willingness-to-pay (WTP) threshold consistent with current health economic standards.^18^

### Sensitivity Analysis

Due to limited data on the response and relapse rates in pouchitis and the variability in the rates observed in RCTs and real-life observational studies, we performed extensive 1-way sensitivity analyses for all probability estimates, ranging from 0% to 99%. Since costs of drugs vary significantly between third-party payers, we also performed 1-way sensitivity analyses for all drug costs. To account for interindividual variabilities in probabilities of response and relapse, cost of drugs and utilities of different health states, we conducted a Monte Carlo analysis with 1000 simulated patients assuming a triangular distribution for all variables. Base-case, minimum, and maximum values used in Monte Carlo analysis are listed in Tables 1 and 2. We also used the results of the Monte Carlo analysis to generate a cost-effectiveness acceptability curve with WTP thresholds ranging from $0 to $500,000 per QALY. Lastly, we also performed sensitivity analysis by changing the follow up time of base case cohorts in both primary and secondary prophylaxis models to 1 year and 5 years.

## Results

### Primary Prophylaxis

#### Base-case analysis

**Figure 2:**
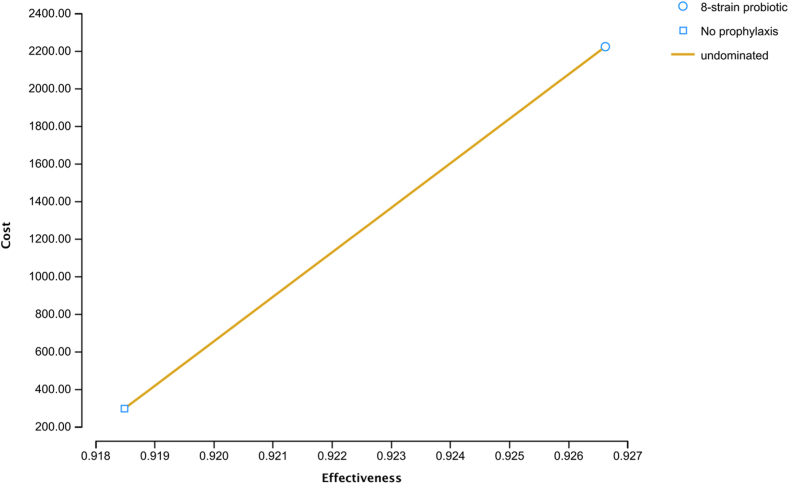
Base case analysis for primary prevention of pouchitis with the 8-strain probiotic compared with no prophylaxis over 2 years.

Base-case analysis showed that over a period of 2 years, prophylaxis with the 8-strain probiotic was more effective (cumulative QALYs: 0.927 vs 0.918) and more expensive (cumulative cost: $2223 vs $299) than no prophylaxis. The ICER of the 8-strain probiotic prophylaxis was $236,076/QALY, implying that no prophylaxis was more cost-effective that the 8-strain prophylaxis for primary prevention of pouchitis over 2 years with a WTP threshold of $100,000/QALY (Figure 2). The 8-strain probiotic prophylaxis was also not cost-effective over 1-year and 5-year time periods with ICERs of $236,706 and $240,690/QALY, respectively. Additional base-case analysis performed assuming empirical antibiotic treatment of the initial episode of pouchitis also showed that the 8-strain probiotic prophylaxis strategy was more effective (cumulative QALYs 0.927 vs 0.919) and more expensive (cumulative cost $2183 vs $155) but overall, not cost effective with an ICER of $257,353/QALY compared with no prophylaxis.

Markov cohort analysis showed that at the end of 2 years, 2% of the base case cohort on the probiotic prophylaxis and 7.3% on no prophylaxis had nonresponse or loss of response to single antibiotic regimens and were treated with a combination of ciprofloxacin and tinidazole where our model terminated. Extending the Markov cohort further to include nonresponse to the dual antibiotic regimen, wherein the patients were treated for CARP with VDZ as the first and IFX as the second line biologic agent, did not significantly change the overall results and no prophylaxis remained the more cost-effective strategy (Table A1).

#### Base-case 1-way sensitivity analysis

We identified a few thresholds on 1-way sensitivity analysis that affected the cost-effectiveness analysis results (Table 3). If the annual probability of developing first episode of pouchitis with no prophylaxis was higher than 76% (base case value 27.8%), the 8-strain probiotic prophylaxis became a cost-effective strategy. The 8-strain probiotic prophylaxis also became cost effective if the 2-week cost of the probiotic decreased to <$41 (base case value $87.2). Other changes in the probability estimates, costs and utilities did not impact the results.

**Table 3:** Results of the 1-Way Sensitivity Analysis of Primary Prophylaxis of Pouchitis

Variable	Base case value	Range	Result
Probability of first pouchitis episode in 1 y on the 8-strain probiotic	10%	0%–100%	• Probiotic prophylaxis was not cost effective at any probability
Probability of first pouchitis episode in 1 y on no prophylaxis	40%	0%–100%	• Above 76%, prophylaxis with the 8-strain probiotic became cost effective
Probability of recurrent pouchitis within 1 y of index pouchitis episode	39%	0%–100%	• Prophylaxis with the 8-strain probiotic was not cost-effective at any probability
Probability of response to ciprofloxacin 500 mg twice daily for 2 wk	77%	0%–100%	• Prophylaxis with 8-strain probiotic was not cost-effective at any probability
Probability of response to metronidazole 500 mg twice daily for 2 wk	70%	0%–100%	• Prophylaxis with 8-strain probiotic was not cost-effective at any probability
Cost of the 8-strain probiotic formulation for 2 wk	$87.2	$1–1000	• At cost less than $42, probiotic prophylaxis became cost effective
			• At cost less than $9, probiotic prophylaxis became the dominant strategy
Cost of ciprofloxacin 500 mg twice daily for 2 wk	$12	$1–100	• Prophylaxis with the 8-strain probiotic was not cost-effective at any cost
Cost of metronidazole 500 mg twice daily for 2 wk	$22.68	$1–100	• Prophylaxis with the 8-strain probiotic was not cost-effective at any cost
Cost of ciprofloxacin 500 mg twice daily and tinidazole 500 mg twice daily for 4 wk	$270	$1–1000	• Prophylaxis with the 8-strain probiotic was not cost-effective at any cost
Cost of first episode of pouchitis	$612.6	$50–10,000	• At cost above $3,935, prophylaxis with the 8-strain probiotic became cost effective
Annual utility of active pouchitis	0.46	0–1	• Prophylaxis with the 8-strain probiotic was not cost-effective at any utility value
Annual utility of no pouchitis	0.68	0–1	• Prophylaxis with the 8-strain probiotic was not cost-effective at any utility value

#### Monte Carlo analysis

On Monte Carlo analysis, the 8-strain probiotic prophylaxis was cost-effective in only 22.9% of simulations (ICER >100,000 in 77% and inferior in 0.1%) compared with no prophylaxis over 2 years (Figure A3). Cost-effectiveness acceptability curves showed that with an increasing WTP threshold, the probiotic prophylaxis became cost-effective in an increasingly higher proportion of trials, ranging from 22.9% at WTP threshold of $100,000 to 94% at WTP threshold of $500,000 (Figure A4).

### Secondary Prophylaxis

#### Base-case analysis

**Figure 3:**
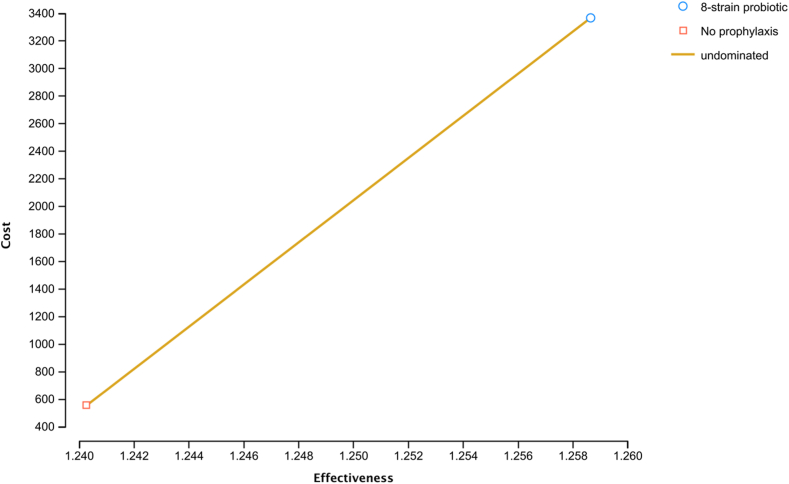
Base case analysis for prevention of pouchitis relapse in infrequent pouchitis with the 8-strain probiotic compared with no prophylaxis over 2 years.

On base-case analysis for prevention of infrequent recurrent pouchitis over a period of 2 years, the 8-strain probiotic prophylaxis was more effective (cumulative QALYs: 1.26 vs 1.24) and more expensive (cumulative cost: $3370 vs $557) compared with no prophylaxis. The ICER for the 8-strain probiotic prophylaxis was $153,011/QALY, implying that no prophylaxis was more cost-effective for patients with infrequent relapsing pouchitis over 2 years with a WTP threshold of $100,000/QALY (Figure 3). The 8-strain probiotic prophylaxis was also not cost-effective over 1-year and 5-year time periods (ICERs $159,529 and $149,706/QALY, respectively).

#### Base-case 1-way sensitivity analysis

On 1-way sensitivity analysis, the 8-strain probiotic became cost effective if the annual probability of pouchitis relapse without prophylaxis was ≥48% (Table 4). If this probability increased further to >99%, the probiotic prophylaxis became the dominant strategy suggesting that the probiotic prophylaxis was cost-effective in patients with experience frequent pouchitis relapses (≥2 episodes/year). The 8-strain probiotic prophylaxis was also cost effective if the cost of 4 weeks of 8-strain probiotic was less than $121 (base case value $175) and if the probability of response to ciprofloxacin in pouchitis was <45%. Other 1-way sensitivity analyses did not impact the results.

**Table 4:** Results of the 1-Way Sensitivity Analysis of Prevention of Pouchitis Relapse in Recurrent Pouchitis

Variable	Base case value	Range	Result
Probability of pouchitis relapse in 1 y on the 8-strain probiotic	14%	0%–100%	• Prophylaxis with the 8-strain probiotic was not cost effective in infrequent pouchitis at any probability
Probability of pouchitis relapse in 1 y on no prophylaxis	39%	0%–100%	• Above 48%, prophylaxis with the 8-strain \was cost effective
			• Above 99%, prophylaxis with the 8-strain probiotic was dominant
Probability of response to ciprofloxacin 500 mg twice daily for 4 wk	77%	0%–100%	• Below 45%, prophylaxis with the 8-strain probiotic was cost-effective
Probability of response to metronidazole 500 mg twice daily for 4 wk	70%	0%–100%	• Prophylaxis with the 8-strain probiotic was not cost-effective at any probability
Probability of response to VDZ		0%–100%	• Prophylaxis with the 8-strain probiotic was not cost-effective at any probability
Probability of loss of response to VDZ in 1 y	40%		• Prophylaxis with the 8-strain probiotic was not cost-effective at any probability
Cost of the 8-strain probiotic for 4 wk	$175	$1–$1000	• At $121 or less, prophylaxis with the 8-strain probiotic was cost effective
			• At $19 or less, prophylaxis with the 8-strain probiotic became the dominant strategy
Cost of ciprofloxacin 500 mg twice daily for 4 wk	$24	$1–$100	• Prophylaxis with the 8-strain probiotic was not cost-effective at any cost
Cost of metronidazole 500 mg twice daily for 4 wk	$45.4	$1–$100	• Prophylaxis with the 8-strain probiotic was not cost-effective at any cost
Cost of ciprofloxacin 500 mg twice daily and tinidazole 500 mg twice daily for 4 wk	$270	$1–$1000	• Prophylaxis with the 8-strain probiotic was not cost-effective at any cost
Cost of 300 mg IFX dose	$987	$1–$5000	• Prophylaxis with the 8-strain probiotic was not cost-effective at any cost
Cost of 300 mg VDZ dose	$6803	$1–$20,000	• Prophylaxis with the 8-strain probiotic was not cost-effective at any cost
Annual utility of active pouchitis	0.46	0–1	• Below 0.21, prophylaxis with the 8-strain probiotic was cost-effective
Annual utility of no pouchitis	0.91	0–1	• Prophylaxis with the 8-strain probiotic was not cost-effective at any probability

#### Monte Carlo analysis

Over a period of 2 years, the 8-strain probiotic prophylaxis was cost-effective in only 2.9% of simulations. It was not cost-effective in 84.4% simulations and was inferior to no prophylaxis in 12.7% (Figure A5). With increasing WTP thresholds, the 8-strain probiotic prophylaxis became cost-effective in an increasingly higher proportion of simulations, from 2.9% at a WTP threshold of $100,000 to 61.9% at a WTP threshold of $500,000 (Figure A6).

## Discussion

In this comprehensive cost-effective analysis of the 8-strain probiotic for primary and secondary prevention of pouchitis compared with no prophylaxis, we made several interesting observations. First, in patients who underwent IPAA for UC, the 8-strain probiotic was not cost-effective for primary prevention of development of pouchitis. Second, in patients who experience infrequent episodes of relapsing pouchitis, the 8-strain probiotic was not cost-effective for preventing relapse of pouchitis. However, it was cost-effective for preventing relapse of pouchitis in patients who experience frequent episodes of pouchitis. These findings can supplement the recent AGA clinical guidelines on management of pouchitis, and facilitate decision-making for patients, providers and payers.^11^

For primary prevention of pouchitis, the 8-strain probiotic was more effective but considerably more costly and overall, not cost effective compared with no prophylaxis. These results were influenced heavily by the cost of the 8-strain probiotic and the probability of first episode of pouchitis without prophylaxis. Despite a much higher proportion of the base case cohort without prophylaxis experiencing pouchitis over 2 years compared with those on probiotic prophylaxis (64% vs 19%) and incurring the additional cost of workup and treatment of pouchitis, probiotic prophylaxis remained almost 10 times more costly than no prophylaxis. The cost of the 8-strain probiotic contributed around 95% to the cumulative cost of the probiotic prophylaxis strategy and around 50% reduction in its cost could make probiotic prophylaxis cost-effective. Probiotic prophylaxis could also be cost effective if the annual risk of pouchitis was higher at ≥72.5% than 40% used in this analysis. This suggests that the 8-strain probiotic could conceivably be cost effective for primary prevention of pouchitis in patients who are at higher than average risk for developing pouchitis, like patients with primary sclerosing cholangitis (PSC), who have 4.2 times higher odds of developing pouchitis compared with patients without PSC.^19^ However, further research is warranted on the effectiveness of the 8-strain probiotic for primary prevention of pouchitis in patients with UC-IPAA and PSC. In the recent clinical guidelines on management of pouchitis, the AGA made no recommendations in favor of, or against the use of the probiotics for primary prevention of pouchitis, trying to balance the high observed efficacy of probiotics, with the high burden of daily probiotic use, to prevent a relatively infrequent and easy to treat event, the first episode of pouchitis.^11^ However, the guidelines did not incorporate a formal cost-effectiveness analysis in determining the final recommendation. Our study provides the cost-effectiveness context to the use of the 8-strain probiotic in this setting.

In evaluating cost-effectiveness of the 8-strain probiotic for prevention of pouchitis relapse in patients who experience infrequent episodes of pouchitis, we used a 39% probability of relapse based on data from a large administrative claims data-based study.^1^ In this setting, the probiotic prophylaxis was marginally more effective but 6-times more costly compared with no prophylaxis, making no prophylaxis more cost-effective. In our model, pouchitis relapse occurred in 26% of patients receiving probiotic prophylaxis compared with 73% of those not receiving prophylaxis over 2 years. Despite a higher cost related to treatment of acute pouchitis and downstream management of CARP in the cohort without prophylaxis, the cumulative cost of daily probiotic use made the probiotic prophylaxis overall significantly more costly. As such, a 30% reduction in the cost of the 8-strain probiotic could make the probiotic prophylaxis cost-effective.

One-way sensitivity analysis of the base case of infrequent relapsing pouchitis showed that the 8-strain probiotic prophylaxis was cost effective if the annual probability of pouchitis relapse was ≥ 48% and was dominant at annual probability of pouchitis relapse >99%. This suggests that in patients with relapsing pouchitis who experience ≥1 relapses per year, the probiotic prophylaxis can be less costly and more effective than no prophylaxis. While these results are based on the high efficacy of the 8-strain probiotic in preventing pouchitis relapse observed in RCTs, it is important to note that anecdotal clinical experience and observational data suggest that the probiotic prophylaxis may not be as effective in real life. For example, one observational study showed that only 20% patients remained on the probiotic without experiencing pouchitis relapse after a median of 8 months.^20^ If the effectiveness of the 8-strain probiotic prophylaxis was truly this low in real life, it would not be cost-effective for either infrequent or frequent relapsing pouchitis. The recent AGA guidelines on management of pouchitis suggest using probiotics for preventing recurrent episodes of pouchitis with a caveat that those who experience infrequent episodes may choose to avoid secondary prevention strategies. Our findings supplement the guidelines by confirming that the 8-strain probiotics can be cost effective in frequent relapsing pouchitis but not in infrequent relapsing pouchitis.

Our study has several strengths. We performed a comprehensive evaluation of the existing literature and a meta-analysis to derive the probabilities of relapse and response. We used a third-party payers’ perspective in this analysis, which is the most appropriate approach in the United States since third-party payers determine the cost of diagnostic and therapeutic interventions. We used Medicare Part D and B program data to derive the costs of drugs as they reflect the actual cost that various buyers pay the manufacturers to purchase drugs. Moreover, since Medicare is the largest third-party payer in the United States, commercial third-party payers tend to follow Medicare’s cost reimbursement plan.

We also acknowledge some limitations of our study. While we used the best available data on transition probabilities between different health states, we also made certain assumptions, particularly related to the drug sequencing, in the pouchitis management algorithm. However, we tried to ensure that the assumptions that we made were pragmatic and consistent with real-life treatment approach. Nevertheless, it is conceivable that variations in the drug sequencing may impact the cost-effectiveness analysis results. In primary and secondary prophylaxis models, we assumed 100% response to combination antibiotic therapy and IFX, respectively, as terminal events. While these assumptions aren’t consistent with real-life pouchitis treatment responses, we conducted additional analysis to evaluate the potential impact of these assumptions on our results. In the primary prophylaxis model, extending the analysis to include treatment of pouchitis refractory to combination antibiotic therapy with VDZ and IFX as the first- and second-line advanced therapies did not change the overall base case analysis results. In the secondary prophylaxis model, Markov cohort analysis showed that only 0.4% of patients on probiotic prophylaxis and 1.1% of patients without any prophylaxis reached the terminal event in 2 years, suggesting that the impact of our assumption on the overall analysis would be minimal. Though our results rely on the best available data on the effectiveness of different prevention and treatment strategies for pouchitis, we recognize that data largely comes from small studies and real-life differences in their effectiveness could impact the overall results. This is also true for the probability estimates for development of acute pouchitis, recurrent pouchitis and CARP used in the analysis that were derived from limited existing literature. Lastly, since our analysis was conducted in the US health-care context, its results may not apply to other countries with different health economic landscapes.

## Conclusion

Based on the existing data, primary prophylaxis with the 8-strain probiotic formulation may not be cost-effective in patients with UC who undergo IPAA surgery. The use of the 8-strain probiotic for prevention of pouchitis relapses may also not be cost effective in patients who experience infrequent relapses but might be cost-effective in those who experience frequent relapses. These results will help clinicians in the management of patients with pouchitis, and payers and policymakers in determining the optimal allocation of health-care resources.

## Supplementary Data

Supplementary Material

Extended PDF
