# Supplementary material for: Cost Effectiveness of the 8-Strain Probiotic in Primary and Secondary Prophylaxis of Pouchitis
Source: Gastro Hep Adv. 2025 Aug 28;5(1):100776. doi: 10.1016/j.gastha.2025.100776 (PMC12546965; doi:10.1016/j.gastha.2025.100776)

Supplementary Material

Supplemental Table 1 – Base case analysis for primary prophylaxis of pouchitis with extension of the model to include treatment of chronic antibiotic refractory antibiotic

	Cumulative cost ($)	Cumulative effectiveness (QALY)	ICER ($/QALY)
No prophylaxis	604	0.917	Reference
8-strain probiotic	2301	0.926	181,455

Supplemental figure 1 – Truncated decision tree for primary prophylaxis of pouchitis with the 8-strain probiotic prophylaxis and no prophylaxis

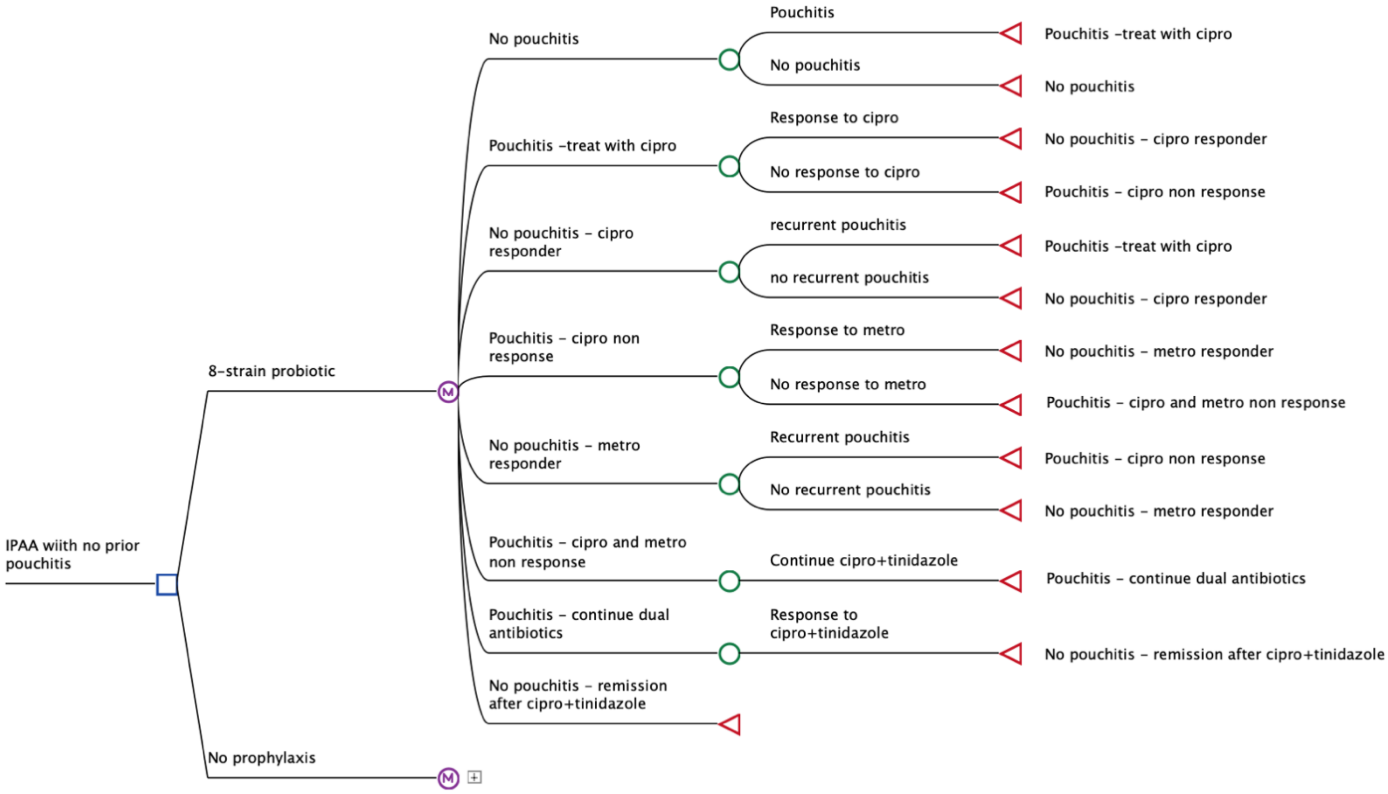

Supplemental figure 2 – Truncated decision tree for prevention of pouchitis relapse with the 8-strain probiotic and no prophylaxis

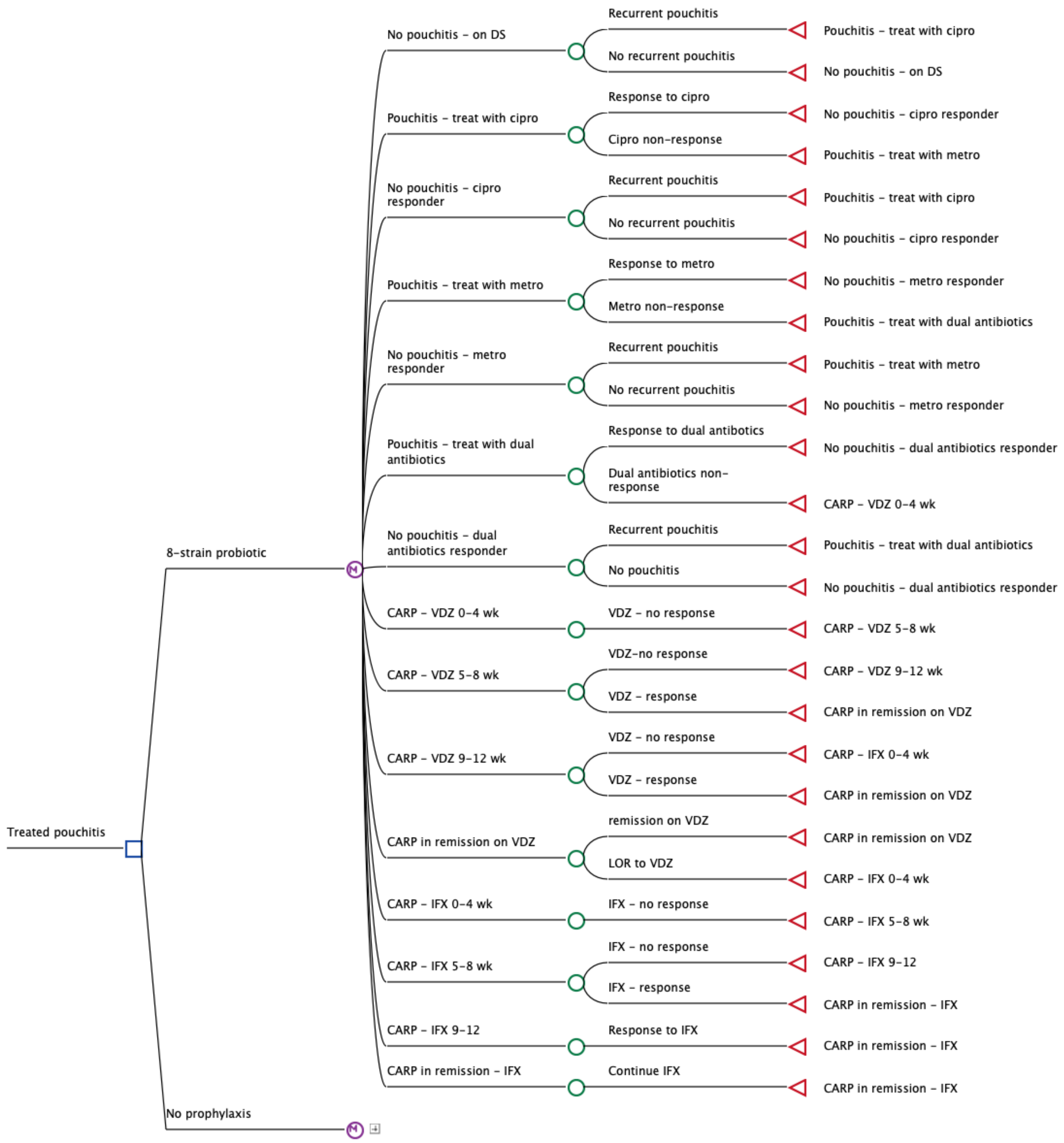

Supplementary Figure 3 – Monte Carlo analysis comparing the 8-strain probiotic with no prophylaxis for primary prevention of pouchitis over 2 years

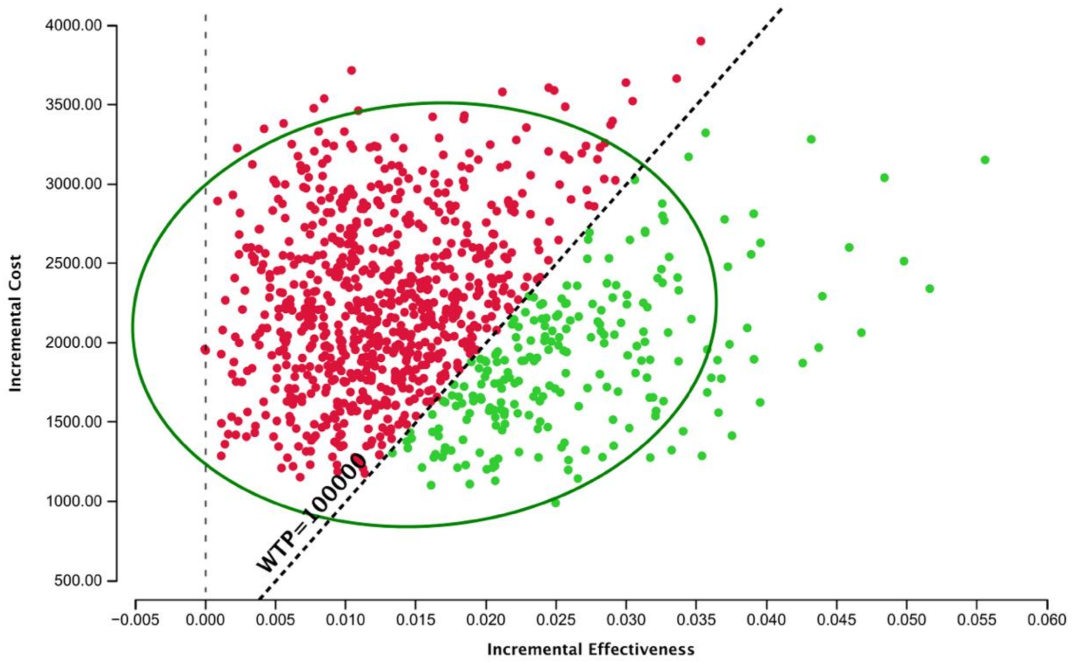

Supplementary figure 4 – Cost effectiveness acceptability curve of the 8-strain probiotic prophylaxis compared with no prophylaxis for primary prevention of pouchitis over 2 years

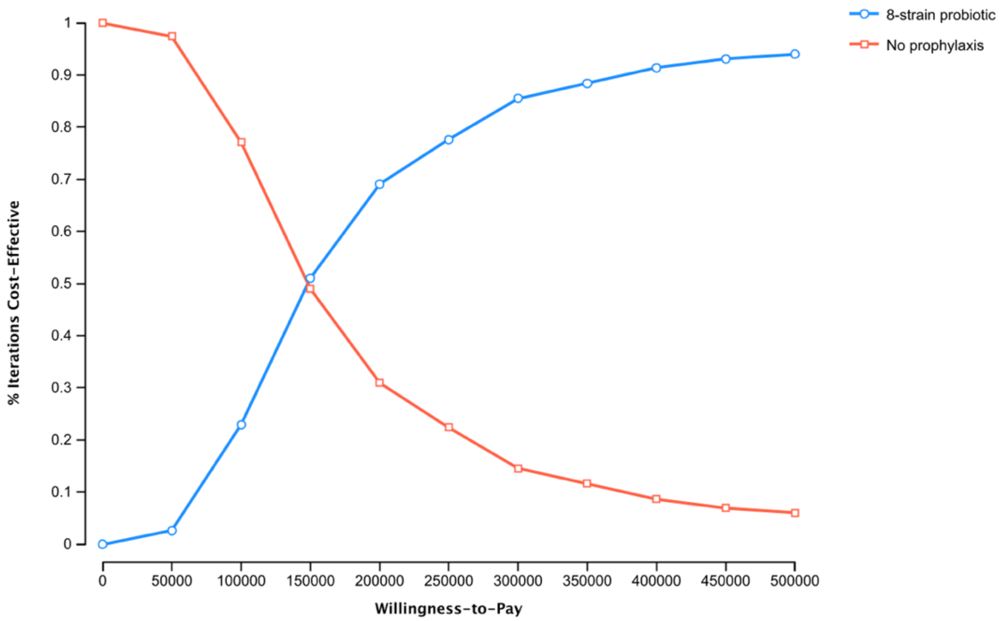

Supplementary Figure 5 – Monte Carlo analysis comparing the 8-strain probiotic with no prophylaxis for prevention of pouchitis relapse in infrequent pouchitis over 2 years

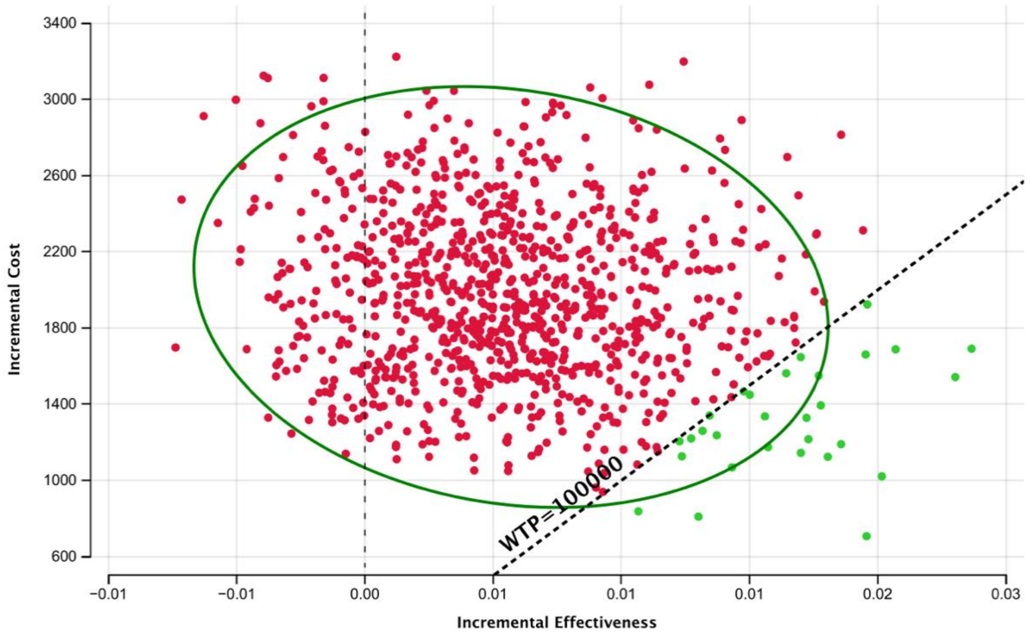

Supplementary figure 6 – Cost effectiveness acceptability curve of the 8-strain probiotic prophylaxis compared with no prophylaxis for prevention of pouchitis relapse in infrequent pouchitis over 2 years

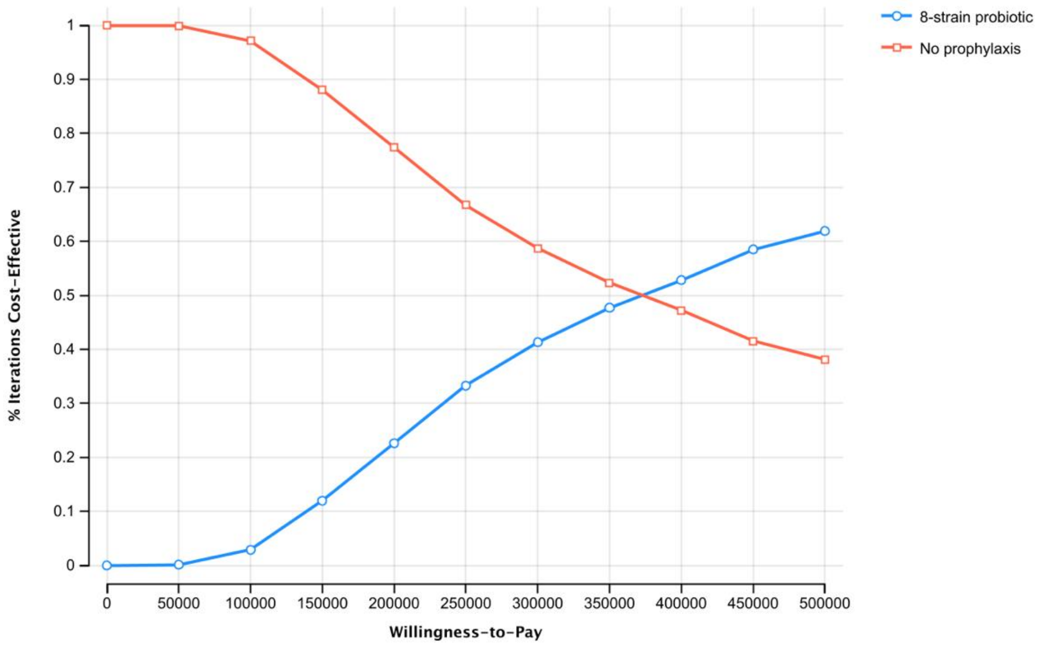

Supplement: Supplementary Material [file mmc1.pdf]
